# Supplementary figures and images for: A Multibreed Genome-Wide Association Study for Cattle Leukocyte Telomere Length
Source: Genes (Basel). 2023 Aug 7;14(8):1596. doi: 10.3390/genes14081596 (PMC10454124; doi:10.3390/genes14081596)

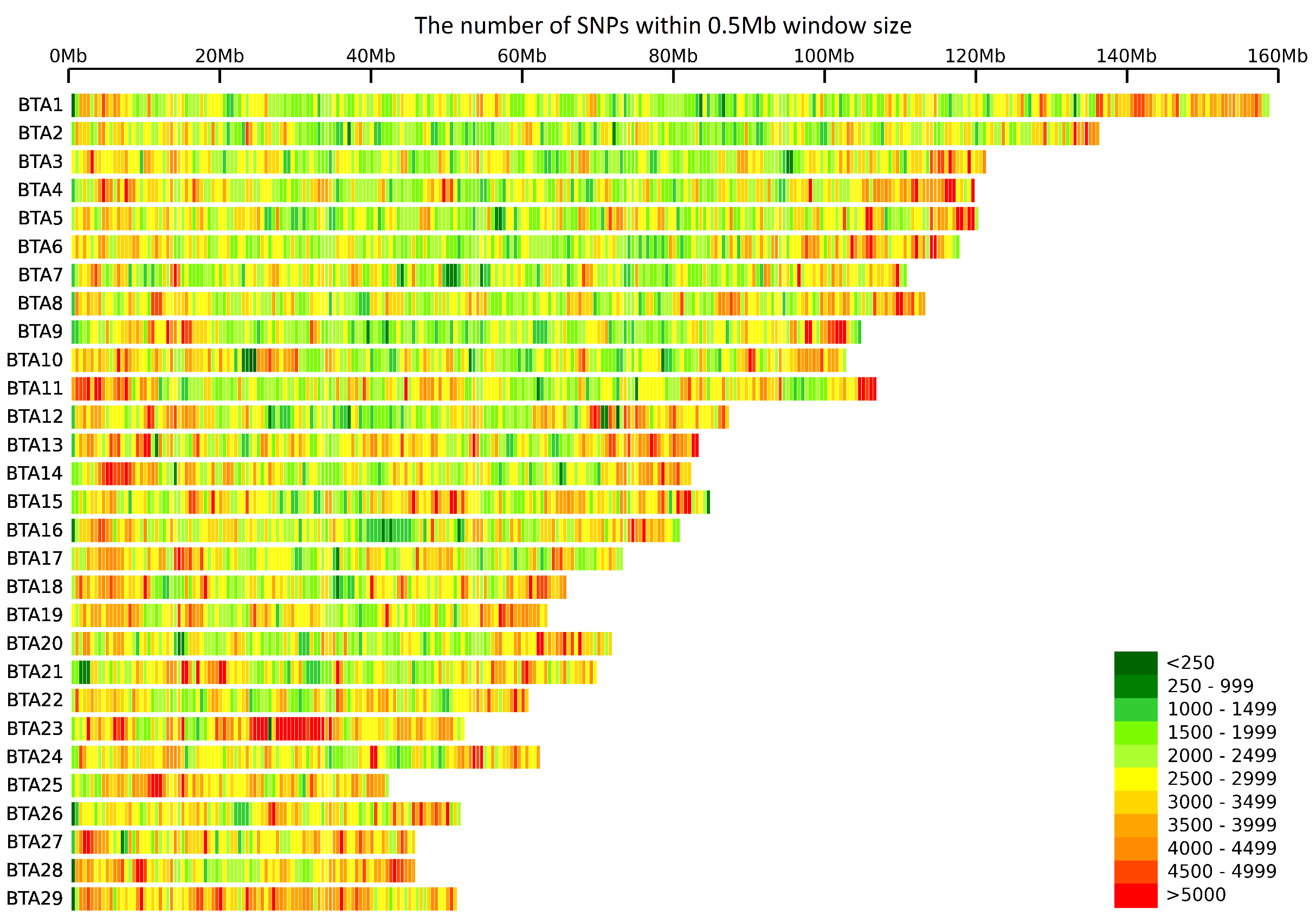

Supplement: Supplementary file 1 [file genes-14-01596-s001.zip › Figure S1.tiff]

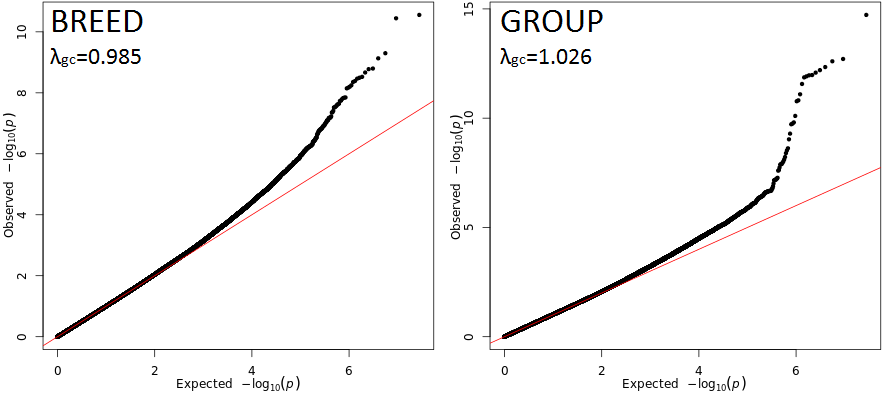

Supplement: Supplementary file 1 [file genes-14-01596-s001.zip › Figure S2.tif]
